# Synthesis and Characterization of New 3-(4-Arylpiperazin-1-yl)-2-hydroxypropyl 4-Propoxybenzoates and Their Hydrochloride Salts

**DOI:** 10.3390/molecules21060707

**Published:** 2016-06-01

**Authors:** Pavlina Marvanova, Tereza Padrtova, Tomas Pekarek, Jiri Brus, Jiri Czernek, Petr Mokry, Otakar Humpa, Michal Oravec, Josef Jampilek

**Affiliations:** 1 Department of Chemical Drugs, Faculty of Pharmacy, University of Veterinary and Pharmaceutical Sciences, Palackeho 1, 61242 Brno, Czech Republic; marvanovap@gmail.com (P.M.); padrtova.tereza@seznam.cz (T.P.); 2 Zentiva k.s., U kabelovny 130, 10237 Prague 10, Czech Republic; tomas.pekarek@atlas.cz; 3 Institute of Macromolecular Chemistry, Academy of Sciences of the Czech Republic, Heyrovsky sq. 2, 16206 Prague 6, Czech Republic; brusjiri@seznam.cz (J.B.); czernek@imc.cas.cz (J.C.); 4 CEITEC—Central European Institute of Technology, Masaryk University, Kamenice 753/5, 62500 Brno, Czech Republic; otakar.humpa@ceitec.muni.cz; 5 Global Change Research Institute CAS, Belidla 986/4a, 60300 Brno, Czech Republic; oravec.m@czechglobe.cz; 6 Department of Pharmaceutical Chemistry, Faculty of Pharmacy, Comenius University, Odbojarov 10, 83232 Bratislava, Slovakia

**Keywords:** arylcarbonyloxyaminopropanols, phenylpiperazines, synthesis, CP/MAS NMR spectroscopy, IR spectroscopy, principle components analysis

## Abstract

Five new 3-(4-arylpiperazin-1-yl)-2-hydroxypropyl 4-propoxybenzoates were designed and synthesized as potential dual antihypertensive agents. The compounds were prepared as free bases and subsequently transformed to hydrochloride salts. The position of protonation of nitrogen atoms in the piperazine ring of hydrochloride salts was determined by means of ^13^C-CP/MAS and ^15^N-CP/MAS NMR and IR spectroscopy. Using these solid-state analytical techniques, it was found that both nitrogen atoms were protonated when excess hydrogen chloride was used for preparation of salts. On the other hand, when the equimolar amount of hydrogen chloride was used, piperazine nitrogen substituted by aryl was protonated.

## 1. Introduction

Antagonists of β-adrenergic receptors, so-called β-blockers, are essential drugs that have been widely used in the treatment of many cardiovascular and non-cardiovascular diseases for more than 50 years. They have played an important role in the treatment of arterial hypertension, cardiac arrhythmias, heart failure, ischemic heart disease, glaucoma, tremor, thyrotoxicosis and anxiety [1,2]. The latest generation of β-blockers commonly possesses some additional beneficial features that can improve their therapeutic profile and reduce their sometimes serious side effects. These features are mainly antioxidant properties (e.g., carvedilol [3]), vasodilatory activity (e.g., carvedilol, nebivolol [3,4]) and ultra-short effect (e.g., esmolol, landiolol [5,6]).

The studied compounds, salts of 3-(4-arylpiperazin-1-yl)-2-hydroxypropyl 4-propoxybenzoates, were designed as soft analogues of aryloxyaminopropanols [7] in accordance with the concept of soft drugs proposed by N. Bodor [8]. Soft drugs are designed highly effective agents with predictable and controllable one-step *in vivo* biotransformation to non-toxic and inactive products after they have achieved their desired pharmacological effect, *i.e.*, the molecule is deactivated and detoxified shortly after it has exerted its biological effect. Frequently, a metabolically sensitive spot (critical structural feature, e.g., ester moiety) is incorporated into the structure of approved drugs, and in the spot they are metabolized and inactivated [7,8].

This contribution is focused on the synthesis and exact determination of the structure parameters of five new 3-(4-arylpiperazin-1-yl)-2-hydroxypropyl 4-propoxybenzoates. Compounds were prepared as hydrochloride salts to increase their solubility in water. The position of protonation of nitrogen atoms in the piperazine ring of hydrochloride salts was determined by means of ^13^C-CP/MAS and ^15^N-CP/MAS NMR spectroscopy and IR spectroscopy. The cardiovascular activity of the discussed compounds is provided by the presence of the arylcarbonyloxyaminopropanol pharmacophore (β-adrenolytic activity; the ester moiety ensures ultra-short effect) and the phenylpiperazine fragment (α_1_-adrenolytic activity) [2,8]. The arylcarbonyloxyaminopropanol scaffold is formed by the benzene ring (as a part of 4-hydroxybenzoic acid) substituted at the C_(4)_ position with a propoxy chain. The phenylpiperazine segment, the basic part of the molecule, is either unsubstituted or substituted with a methoxy or a fluoro moiety in the C_(2)_′ or C_(4)_′ position. Drugs used in clinical practice [3,4,5,6] as well as investigational agents [9,10,11] are characterized by similar structural features [2]; nevertheless, the biological effects of the discussed salts of 3-(4-arylpiperazin-1-yl)-2-hydroxypropyl 4-propoxybenzoates have not been determined so far.

## 2. Results and Discussion

### 2.1. Chemistry

**Scheme 1:**
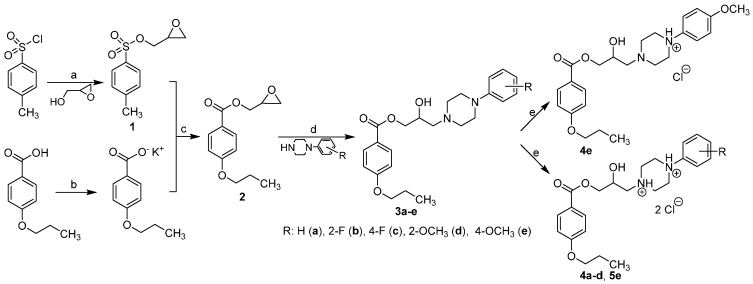
Synthesis of discussed compounds **1**–**5e**. *Reagents and conditions*: (**a**) TEA, DCM, 0 °C; (**b**) KOH, MeOH, IPA; (**c**) DMF, reflux; (**d**) arylpiperazine, IPA, reflux; (**e**) HCl, Et_2_O.

The final substances were prepared by four-step synthesis as described in Scheme 1. Tosylate intermediate **1** was prepared according to the published procedure, and the spectroscopic and physical data were in agreement with those reported in the literature [12]. It was necessary to maintain the temperature at 0 °C to obtain good yields. Raising the temperature or prolonging the reaction lowered the yield of the reaction dramatically. Intermediate **2** was already described in the literature but was prepared by a different synthetic method, and the spectroscopic data was in agreement with the literature [13]. In the first two steps of the synthesis, the commercially available 4-propoxybenzoic acid was transformed to the potassium salt by potassium hydroxide in a mixture of methanol and propan-2-ol in ratio 1:3. Reaction of tosylate **1** with potassium 4-propoxybenzoate yielded oxiran-2-ylmethyl 4-propoxybenzoate (**2**). The reaction was performed in dimethylformamide for 7 h at 70 °C; changing the solvent (acetone, acetonitrile) or lowering the temperature did not give the desired result. Opening of the oxirane ring of compound **2** with appropriate phenylpiperazine in propan-2-ol for 1 h at 70 °C and for 72 h at ambient temperature resulted in the formation of the target bases. Prolongation of heating by 5 h shortened the reaction time but led to fewer and less pure products. Finally, bases **3a**–**e** were converted to their hydrochloride salts **4a**–**e** and **5e** to enhance their solubility in water.

### 2.2. NMR, CP/MAS Spectra

**Figure 1:**
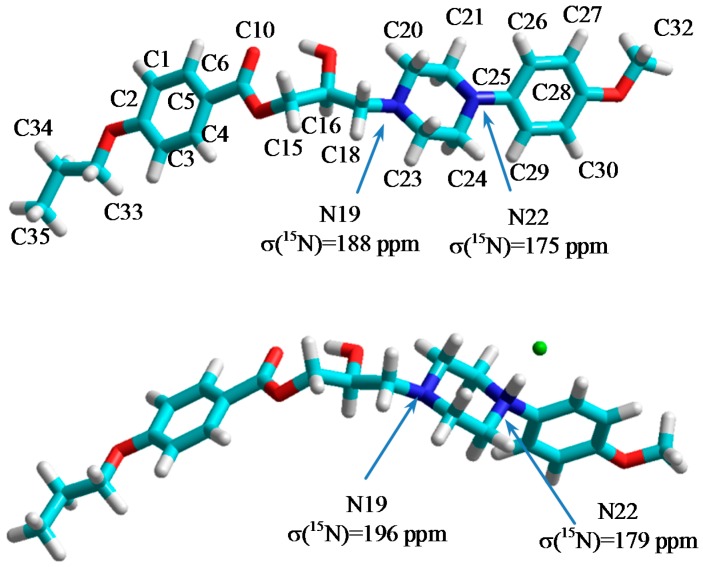
DFT optimized structure of base **3e** (R = 4-OCH_3_) and its predicted DFT optimized monohydrochloride salt **4e** (R = 4-OCH_3_), carbon and nitrogen unit numbering and calculated ^15^N-NMR isotropic shielding parameters σ(^15^N).

In solid state, the assignment of ^15^N-NMR signals is generally complicated by the extremely low sensitivity of ^1^H-^15^N heteronuclear correlation (HETCOR) NMR experiments. This is due to the combination of many factors, among which slow ^1^H spin-lattice relaxation, signal broadening caused by static disorder and large chemical shift anisotropy inducing the formation of intensive spinning side bands play major roles. Consequently, even the measurement of a single 1D ^15^N-CP/MAS NMR spectrum usually requires overnight data acquisition. Therefore, the assignment of ^15^N-CP/MAS NMR signals in the piperazine ring in crystalline systems was based on DFT structure optimization and the subsequent DFT calculation of ^15^N-NMR shielding parameters of compound **3e**; see Figure 1. This approach has been verified in our previous study, where we found out that the agreement between experimental data (^15^N-NMR chemical shifts) and the predicted shielding values is very good, usually not exceeding 3–5 ppm [14]. Consequently, because the difference between the detected ^15^N-CP/MAS NMR signals of N22 and N19 sites is *ca.* 10 ppm, the proposed strategy can be used for interpretation of the recorded spectra. Specifically, for the **3e** compound, the relative difference between the DFT calculated ^15^N-NMR shielding parameters (13 ppm) perfectly agrees with the experimentally determined relative difference (*ca.* 13 ppm). Consequently, the signal at *ca.* 50 ppm was assigned to the N19 site, while the signal at *ca.* 60 ppm was attributed to N22 sites. Moreover, this assignment is in good agreement with the previously published data for piperazine analogues (sildenafil citrate, [15]).

**Figure 2:**
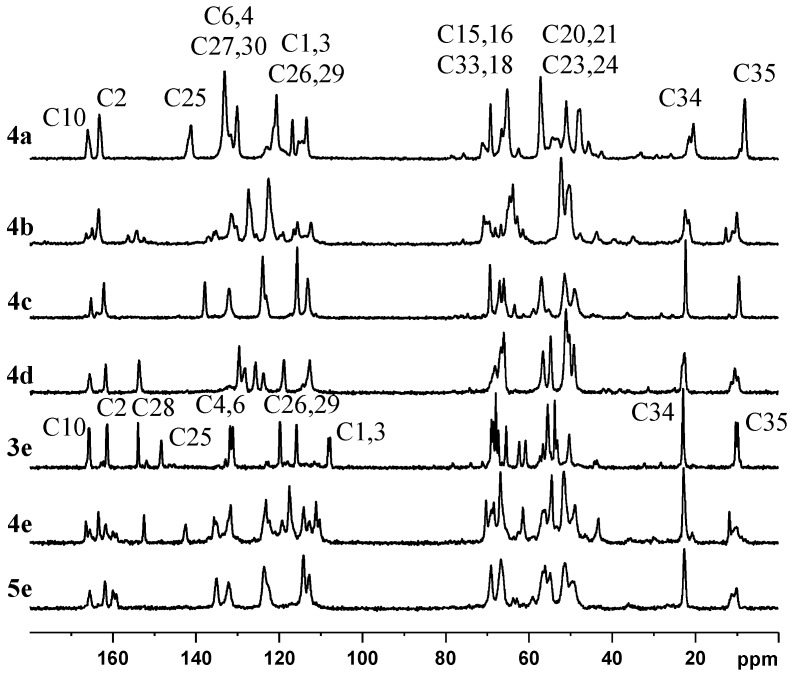
^13^C-CP/MAS NMR spectra of compounds **4a** (R = H), **4b** (R = 2-F), **4c** (R = 4-F), **4d** (R = 2-OCH_3_), **3e** (R = 4-OCH_3_, base), **4e** (R = 4-OCH_3_, eq. HCl) and **5e** (R = 4-OCH_3_, excess of HCl) together with partial illustrative signal assignment.

**Figure 3:**
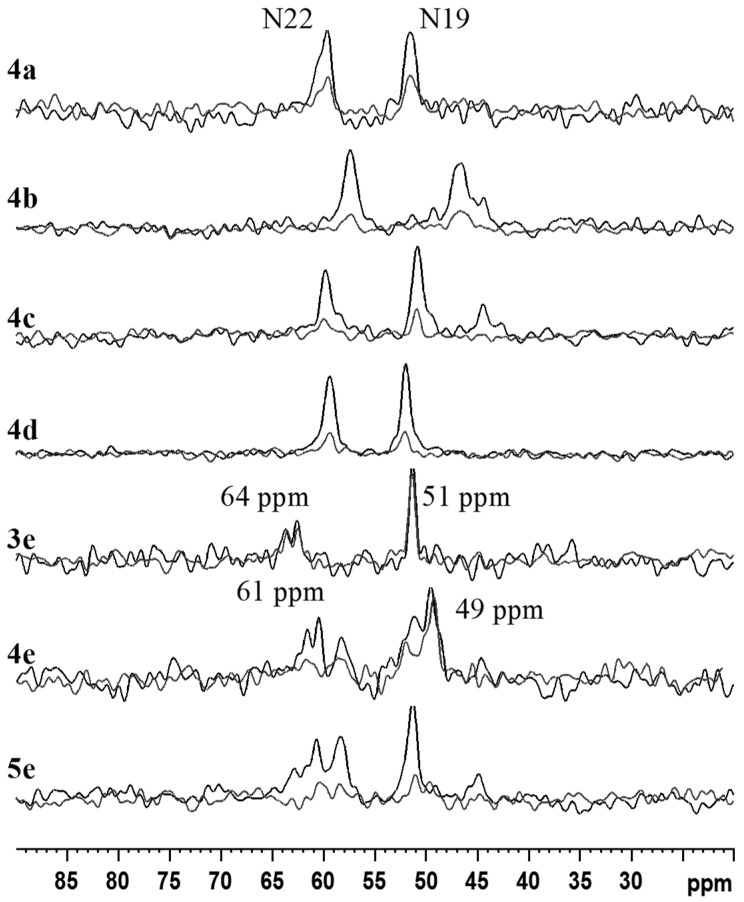
^15^N-CP/MAS NMR and ^15^N-CPPI/MAS NMR (PI = 100 µs) spectra of compounds **4a** (R = H), **4b** (R = 2-F), **4c** (R = 4-F), **4d** (R = 2-OCH_3_), **3e** (R = 4-OCH_3_, base), **4e** (R = 4-OCH_3_, eq. HCl) and **5e** (R = 4-OCH_3_, excess of HCl) together with signal assignment.

The recorded ^13^C- and ^15^N-CP/MAS NMR spectra, in the majority of which splitting and asymmetric broadening of signals is apparent, reflect the extensive conformation variability of the investigated compounds; see Figure 2 and Figure 3. From the set of the investigated compounds, the most uniform molecular arrangements were found for systems **3e** (base, R = 4-OCH_3_) and **4c** (R = 4-F), for which only a slight disorder or negligible contamination by another polymorphic modification were identified. Whereas the unit cell of compound **4c** consists of a single symmetry-independent molecule (single lines are detected), the symmetrical spitting of the majority of ^13^C-CP/MAS NMR signals from system **3e** clearly indicates the existence of two symmetry-independent molecules in the crystal unit. A slightly more complex splitting of ^13^C-CP/MAS NMR signals of methyl and methylene units (C35, C34) indicates the slight conformation disorder of propyl substituents in crystals of **4a** (R = H), **4b** (R = 2-F) and **4d** (R = 2-OCH_3_) compounds. As reflected by single lines detected in the corresponding ^15^N-CP/MAS NMR spectra, the local structure in the vicinity of nitrogen atoms is nearly unaffected by this local disorder and remains uniform in the whole crystal structure. As demonstrated by multiple splitting of signals in ^13^C- and ^15^N-CP/MAS NMR spectra, the most complex crystal structures can be expected for final monohydrochloride **4e** (R = 4-OCH_3_, prepared by the equimolar amount of HCl) and sample **5e** (R = 4-OCH_3_) prepared from base **3e** with excess of HCl. This is probably due to a combination of several influences, including the conformation variability of the propyl chain and the piperazine ring as well as non-uniform protonation of nitrogen sites. The latter phenomenon will be discussed below. In this regard, considering all possible sources of the signal broadening and splitting, we must take into account a possible presence of different enantiomers in the analysed systems. In principle, ^13^C-CP/MAS NMR spectra of different enantiomers usually differ in terms of the positions and intensities of the corresponding resonance frequencies [16]. Consequently, if a racemic mixture is formed, the presence of ”chiral” impurities can be reflected by the additional set of NMR signals. Unfortunately, there is no simple way to unambiguously identify different enantiomers and distinguish between the signals reflecting different polymorphic forms or different enantiomers.

**Figure 4:**
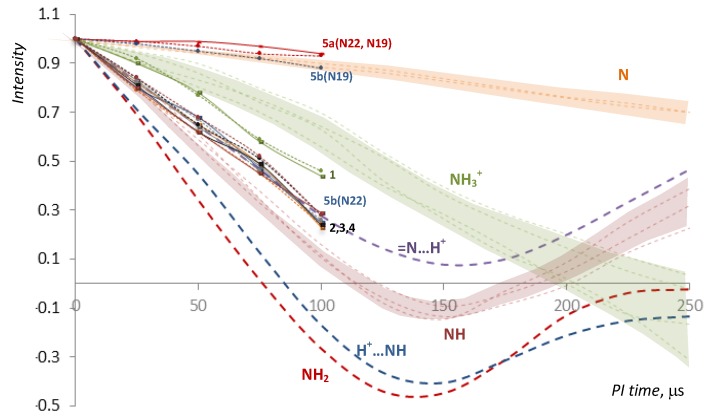
^15^N cross-polarization polarization-inversion profiles. Dots and thin solid lines represent polarization-inversion profiles determined for nitrogen sites N19 and N22 of investigated compounds **4a** (R = H), **4b** (R = 2-F), **4c** (R = 4-F), **4d** (R = 2-OCH_3_), **3e** (R = 4-OCH_3_, base), **4e** (R = 4-OCH_3_, eq. HCl) and **5e** (R = 4-OCH_3_, excess HCl). Thick solid lines represent PI profiles determined for nitrogen moieties (N, NH, =N…H^+^, NH_2_, NH…H^+^ and NH_3_^+^) of model compounds l-His, l-His·HCl·H_2_O, l-Gln, l-Pro and dopamine·HCl.

As indicted by the quantum-chemical calculations demonstrated in Figure 1, the protonation of an –*N*– site in the piperazine ring leads to a decrease in the ^15^N resonance frequencies of both nitrogen sites, regardless which one of them is directly protonated. The calculated changes in ^15^N resonance frequencies are in very good qualitative agreement with the experimental data obtained for base form **3e** and monohydrochloride form **4e**; see Figure 1. However, to verify the protonation of both nitrogen sites and/or to site-specifically locate the position of H^+^ ion, we assessed the strength of ^1^H-^15^N dipolar couplings for each resolved nitrogen site by using the CPPI experiment [17,18,19] with a variable polarization-inversion (PI) period. As was previously demonstrated, the nitrogen moieties N, NH, NH_2_ and NH_3_ exhibit characteristic and clearly distinguishable PI profiles. Moreover, clear differences between the polarization-inversion rates were found for NH and NH^+^ species, reflecting different N-H distances; see Figure 4.

As expected, the insignificant polarization-inversion (PI) decays observed for the base form of **3e** reflect negligible ^1^H-^15^N dipolar couplings involving N22 and N19 sites, thus confirming the absence of H^+^ ion in their vicinity. In contrast, for monohydrochloride compound **4e** prepared from the “equimolar solution”, the flat PI profile was detected for the N19 site only, whereas the nitrogen site N22 exhibits a much stronger ^15^N signal intensity decrease, indicating strong ^1^H-^15^N dipolar coupling. This finding indicates specific protonation of N22 site in compound **4e**. The rate of polarization inversion of N22 (**4e**) corresponds well to the behaviour of ^15^N magnetization of protonated =N…H^+^ sites involved in the charge-transfer assisted hydrogen bonding (e.g., l-His·HCl). The performed DTF geometry optimization of protonated **4e** compound also suggests specific protonation of N22 site. Detailed interpretation and explanation of this finding requires complete x-ray diffraction data and extensive DFT structure optimizations. Such investigation, however, is beyond the aims of this study. The strong hydrogen bonding and protonation of both nitrogen sites N22 and N19 was observed for compound **5e** prepared under excess of HCl. Similar behaviour, *i.e.*, the fast and efficient polarization inversion of ^15^N magnetization of both nitrogen sites N22 and N19, was further observed for compounds **4b**, **4c** and **4d** prepared from the solution with excess HCl. In contrast, a considerably slower decay of ^15^N magnetization of both nitrogen sites N22 and N19 was observed for system **4a**, indicating relatively weak ^1^H-^15^N dipolar couplings. In this compound, we suppose longer N…H^+^ interatomic distances in comparison with the previous discussed compounds **4b**, **4c**, **4d** and **5e**.

### 2.3. IR Data

**Figure 5:**
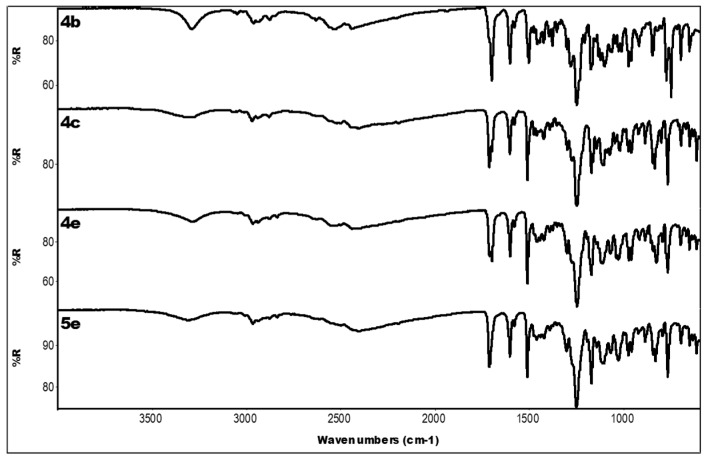
IR spectra of protonated compounds **4b** (R = 2-F), **4c** (R = 4-F), **4e** (R = 4-OCH_3_, equivalent of HCl) and **5e** (R = 4-OCH_3_, excess HCl).

**Figure 6:**
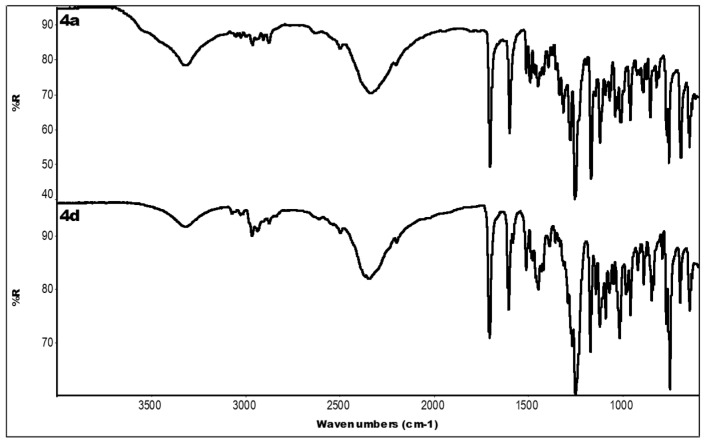
IR spectra of protonated compounds **4a** (R = H) and **4d** (R = 2-OCH_3_).

**Figure 7:**
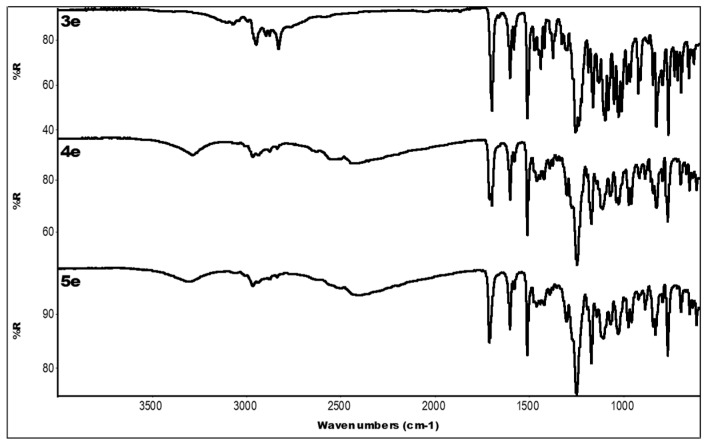
IR spectra of base **3e** (R = 4-OCH_3_) and protonated compounds **4e** (R = 4-OCH_3_, equivalent of HCl) and **5e** (R = 4-OCH_3_, excess HCl).

**Figure 8:**
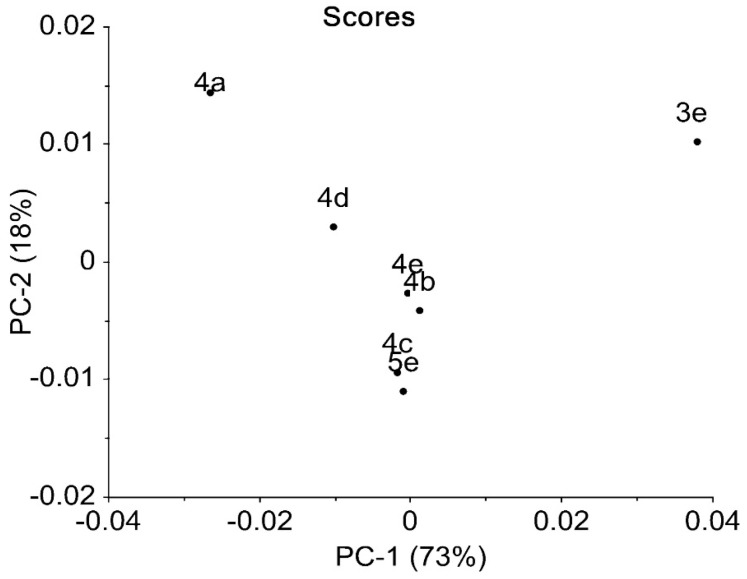
PCA results of IR spectra (reg. 3500–1750 cm^−1^).

**Figure 9:**
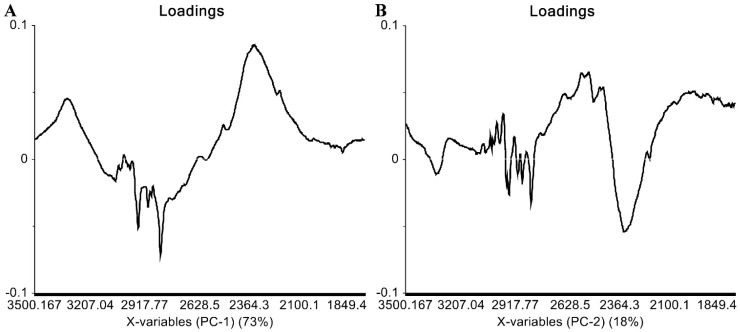
PCA results (loadings graph, PC1) (**A**) and PCA results (loadings graph, PC2) (**B**) of IR spectra (reg. 3500–1750 cm^−1^).

The individual experimental IR data (ATR technique) of all measured compounds **4a**–**d,** **3e**, **4e** and **5e** are summarized in Table 1. The spectra of these samples are presented in Figure 5, Figure 6 and Figure 7. Protonation of at least one nitrogen atom in each of the molecules (except base **3e**) is confirmed by the presence of bands at around 2500–2400 cm^−1^. These bands are assigned to the N-H^+^ stretching vibration. Two groups of spectra of protonated molecules were identified from the comparison of the spectra of compounds **4b**, **4c**, **4e** and **5e** (one group) and **4d** and **4a** (the second group). The spectra are similar to each other in any of these groups. This was confirmed by the principle component analysis (PCA) of the spectra in the region of the largest differences among the spectra (3500–1750 cm^−1^), see Figure 8. The spectrum of base **3e** is separated from all other spectra. It is obvious that the spectra of protonated molecules differ the most in the N-H^+^ vibration region (around 2400 cm^−1^) from the loadings graph for PC1 carrying 73% of explained variance; see Figure 9A. Also PC2 carrying 18% of the explained variance is influenced mainly by the bands assigned to N-H^+^ vibrations at about 2500–2300 cm^−1^; see Figure 9B. Differences in the stretching C-H region (around 2900 cm^−1^) are driven mainly by base molecule **3e**.

All the differences are probably caused mainly by the structural differences in the molecules. As there are no residua of the initial (unprotonated) compound, one can estimate that all molecules were protonated either to mono- or dihydrochloride salt. Infrared spectra are influenced by both chemical and crystalline structure to such an extent that differentiation between particular protonations is not possible.

## 3. Experimental Section

### 3.1. General Information

All reagents were purchased from Sigma-Aldrich in sufficient purity. The solvents were purchased from Lach-Ner and were dried or freshly distilled if necessary. TLC Kieselgel 60 F_254_ plates (Merck, Darmstadt, Germany) visualized by UV irradiation (254 nm) were used to monitor (aceton:toluen 3:1) the reactions and the purity of the prepared substances; the reversed-phase TLC RP-18 F_254_ plates (Merck) were used for the final compounds (0.1 M HCl:aceton 3:2). The melting points were determined on a Kofler hot-plate apparatus HMK (Franz Kustner Nacht KG, Dresden, Germany) and are uncorrected.

The purity of final compounds was analysed by an Agilent 1200 Series system equipped with a DAD system, a quaternary model pump and an automatic injector (Agilent Technologies, Waldbronn, Germany). Data acquisition was performed using the ChemStation chromatography software. The peaks in the chromatogram of the solvent (blank) were deducted from the peaks in the chromatogram of the sample solution. The purity of individual compounds was determined from the area peaks in the chromatogram of the sample solution. The separation was performed on Inertsil^®^ ODS-2 column (GL Sciences Inc., Tokyo, Japan), 3.0 mm × 250 mm, 5 μm, under isocratic conditions. The mixture of acetonitrile-HPLC grade and formate buffer (pH 4.7) in the ratio 60:40 (*v*/*v*) was used as a mobile phase. The total flow rate was 0.4 mL/min; the injection volume was 1 μL; and the column temperature was maintained at 40 °C. The detection wavelength of 254.8 nm was chosen. The time of the analysis was 30 min.

High-resolution mass spectra were measured using acetonitrile hypergrade for LC-MS LiChrosolv^®^ purchased from Merck and acetic acid from Sigma-Aldrich Chemie (Steinheim, Germany). The Purelab Classic (ELGA LabWater, High Wycombe, Bucks, UK) was used to generate high purity water for preparation of aqueous mobile phase. Samples were analysed twice on HPLC-HRMS with negative polarity of MS-Orbitrap. Records from a DAD detector and from a high resolution mass spectrometer were monitored and saved for check of system and evaluation results. For the high performance liquid chromatography part, a Dionex Ultimate 3000 (Thermo Fisher Scientific, West Palm Beach, FL, USA) was used. The column used was a Hypersil Gold column 2.1 mm × 150 mm, 3 μm (Thermo Fisher Scientific). The flow rate of mobile phases was 0.3 mL/min, and column temperature was set at 30 °C. The HPLC mobile phase consisted of (A) acetonitrile and (B) water containing 0.1% acetic acid. Both mobile phases (A) and (B) were filtrated and degassed for 10 min in an ultrasonic bath prior to use. Gradient elution chromatography was performed starting with 10% A and 90% B and held for 5 min. Within a time interval of 5–20 min, A% composition was increased to 90%. This composition was then maintained for 5 min after which the system was equilibrated for 5 min to initial conditions (10% A and 90% B). Wavelengths 254, 272, 274 and 331 nm were monitored. MS and MS^n^ were performed using a LTQ Orbitrap XL™ Hybrid Ion Trap-Orbitrap Fourier Transform Mass Spectrometer (Thermo Fisher Scientific) equipped with a HESI II (Heated electrospray ionization) source. The HRMS Orbitrap was operated in full scan with resolution 60,000. Full scan spectra were acquired over mass range *m*/*z* 65–1000 in the negative mode. The resolution and the sensitivity of the Orbitrap were controlled by an injection of the mixed standard (phenolic compounds) after analysing each 25 samples, and resolution was also checked with the help of lock masses (phthalates). Blanks were also analysed during the sequence. The compounds were confirmed by HPLC/HRMS; high-resolution (5 ppm) and isotopic ratios were used for their identification.

The NMR spectra of compounds **4a**–**e** in the liquid state were measured in DMSO-*d*_6_ solution on a Bruker Avance III HD NMR spectrometer 700 MHz (700.25 MHz for ^1^H and 176.08 MHz for ^13^C nucleus, Bruker BioSpin, Karlsruhe, Germany) equipped with a 5 mm sensitive triple-resonance (^1^H-^13^C-^15^N) cryoprobe optimized for ^13^C detection. Spectra were recorded at the temperature of 30 °C. The chemical shifts δ are reported in ppm relative to tetramethylsilane (TMS), referenced to the chemical shifts of residual solvent resonance (DMSO, 2.5 ppm for ^1^H and 39.5 ppm for ^13^C). The coupling constants (*J*) are reported in Hz. All major signals were assigned on the basis of ^1^H, ^13^C{H}, ^13^C-APT, ^1^H-^1^H COSY, ^1^H-^13^C HSQC and ^1^H-^13^C HMBC experiments. The NMR spectra of some intermediates in the liquid state were measured in DMSO-*d*_6_ on a Gemini-2000 FT-NMR spectrometer (200 MHz for ^1^H and 50 MHz for ^13^C, Varian Comp., Palo Alto, CA, USA).

Solid-state NMR spectra were measured at 11.7 T using a Bruker Avance III HD 500 US/WB NMR spectrometer (Bruker) in 4-mm ZrO_2_ rotors. The ^13^C cross-polarization (CP) magic angle spinning (MAS) NMR spectra were measured at a spinning frequency of 11 kHz, a nutation frequency of B_1_(^13^C) field of 62.5 kHz and a contact time of 1 ms with a repetition delay of 8–12 s. The number of scans providing acceptable signal-to-noise ratio was 1–2 K. The ^15^N-CP/MAS NMR spectra were measured at a MAS frequency of 10 kHz, a nutation frequency of B_1_(^15^N) field of 42 kHz and a contact time of 2 ms with a repetition delay of 8–12 s. The detection of high-quality ^15^N-CP/MAS NMR spectra required the acquisition of 8–20 K scans. As demonstrated previously [17,20], for identification of NH^+^ moiety, the ^1^H-^15^N dipolar profiles were measured using the cross-polarization/polarization-inversion (CPPI) experiment [18]. The CP period was set to 500 µs, and the PI period was incrementally increased from 0 to 100 µs. Glycine was used as an external standard to calibrate ^13^C- and ^15^N-NMR chemical shift scales (176.03 and 35.45 ppm, respectively). All the performed NMR experiments were measured under active cooling. Temperature calibration was performed with Pb(NO_3_)_2_ using a procedure described in the literature [19].

The infrared (IR) spectra were measured on a Nicolet Nexus FT-IR spectrometer (Thermo Fisher Scientific) using ATR (ZnSe) instrumentation. The spectra were obtained by accumulation of 12 scans with 4 cm^−1^ resolution in the region 4000–600 cm^−1^. The instrument was controlled by Omnic v. 8.3 software (Thermo Fisher Scientific). Spectra were treated and principle component analysis (PCA) calculated in the Unscrambler X program v. 10.3. (CAMO, Oslo, Norway).

### 3.2. Synthesis

#### 3.2.1. Synthesis of Intermediates

*Oxiran-2-ylmethyl 4-methylbenzensulfonate* (**1**). *p*-Toluensulfonyl chloride (2.6 g, 0.014 mol) was slowly added to the mixture of (oxiran-2-yl)methanol (1.04 mL, 0.014 mol) and triethylamine (1.84 g, 0.018 mol) in dichlormethane (20 mL) cooled at 0 °C. The mixture was then stirred for 1 h at ambient temperature. The precipitate was filtered, and the organic phase was washed with 2 M HCl, saturated solution of Na_2_CO_3_ and water, dried over MgSO_4_, and then the solvent was evaporated. Product was recrystallized from petroleum ether/diethyl ether in ratio 5:1. Yield: 97%; R_f_: 0.77 (ethyl acetate/petroleum ether 1:1); M.p.: 35–37 °C; ^1^H-NMR (200 MHz, DMSO-*d*_6_) δ: 7.80 (d, *^3^J* = 8.4 Hz, 2H, Ar-SO_2_), 7.49 (d, *^3^J* = 8.4 Hz, 2H, Ar-CH_3_), 4.41 (dd, *^2^J* = 11.5 Hz, *^3^J* = 2.4 Hz, 1H, TsOCH_2_), 3.81 (dd, *^2^J* = 11.5 Hz, *^3^J* = 7.1 Hz, 1H, TsOCH_2_), 3.22–3.14 (m, 1H, CH-oxiran), 2.79–2.74 (m,1H, CH_2_-oxiran), 2.60 (dd, *^2^J* = 5.0 Hz, *^3^J* = 2.6 Hz, 1H, CH_2_-oxiran), 2.42 (s, 3H, CH_3_); ^13^C-NMR (50 MHz, DMSO-*d*_6_) δ: 144.99, 132.20, 130.10, 127.51, 71.58, 48.49, 43.63, 20.98.

*Oxiran-2-ylmethyl 4-propoxybenzoate* (**2**). A mixture of 4-propoxybenzoic acid (5 g, 0.028 mol) in methanol (75 mL) and KOH (2.2 g, 0.042 mol) in propan-2-ol (50 mL) was stirred for 1 h at room temperature, and after that, propan-2-ol (175 mL) was added for the final ratio methanol:propan-2-ol 1:3. The resulting white precipitate was collected by filtration and dried under low pressure. A mixture of appropriate potassium salt (5 g, 0.023 mol) with (oxiran-2-yl)methyl 4-methylbenzensulfonate (**1**, 2.6 g, 0.011 mol) in DMF (27 mL) was heated for 7 h at 70 °C. The reaction was monitored by TLC. The solvent was evaporated, and the residue was dissolved in ethyl acetate and washed with water; the organic layer was dried over MgSO_4_, and ethyl acetate was evaporated. Yield: 83%; R_f_: 0.77 (ethyl acetate/petroleum ether 1:1); ^1^H-NMR (200 MHz, DMSO-*d*_6_) δ: 7.92 (d, *^3^J* = 8.8 Hz, 2H, Ar-COO), 7.04 (d, *^3^J* = 8.8 Hz, 2H, Ar-O), 4.60 (dd, *^2^J* = 12.4, *^3^J* = 2.7 Hz, 1H, COOCH_2_), 4.04 (dd, *^2^J* = 12.4 Hz, *^3^J* = 6.4 Hz, 1H, COOCH_2_), 4.00 (t, *^3^J* = 6.5 Hz, 1H, CH_2_CH_2_), 3.36–3.28 (m, 1H, CH-oxiran), 2.83 (dd, *^2^J* = 5.2 Hz, *^3^J* = 4.3 Hz, 1H, CH_2_-oxiran), 2.72 (dd, *^2^J* = 5.0 Hz, *^3^J* = 2.7 Hz, 1H, CH_2_-oxiran), 1.83–1.65 (m, 2H, CH_2_CH_3_), 0.97 (t, *^3^J* = 7.3 Hz, 3H, CH_3_); ^13^C-NMR (50 MHz, DMSO-*d*_6_) δ: 165.01, 162.68, 131.22, 121.28, 114.35, 69.24, 64.87, 48.97, 43.73, 21.76, 10.10.

#### 3.2.2. Synthesis of Final Compounds

Intermediate **2** (0.5 g, 0.002 mol) was added to the solution of the corresponding amine (0.002 mol) in propan-2-ol (15 mL). The reaction mixture was heated at 80 °C for 1 h and stirred for 72 h at room temperature. After that, the reaction mixture was cooled at least for 24 h at −18 °C. Precipitating crude bases **3a**–**e** were filtered and dissolved in diethyl ether or chloroform and transformed to their dihydrochloride salts **4a**–**d**, **5e** by addition of an excess of saturated ethereal hydrogen chloride. Hydrochloride salt **4e** was prepared using the equimolar amount of ethereal hydrogen chloride. The precipitate was filtered and recrystallized from propan-2-ol, if necessary.

*1-{2-Hydroxy-3-[(4-propoxybenzoyl)oxy]propyl}-4-phenylpiperazinediium dichloride* (**4a**). Yield: 53%; R_f_: 0.87; R_f_(rev): 0.45; M.p.: 208–211 °C; HPLC pur. 99.20% (254.8 nm); ^1^H-NMR (700.25 MHz, DMSO-*d*_6_) δ: 10.83 (bs, 2H, NH^+^), 7.98 (d, *^3^J* = 8.9 Hz, 2H, ArCOO), 7.28–7.25 (m, 2H, Ar), 7.04 (d, *^3^J* = 8.9 Hz, 2H, ArO), 7.02–7.01 (m, 2H, NAr), 6.88–6.86 (m, 1H, Ar), 4.79 (s, 1H, OH), 4.50–4.47 (m, 1H, CH(OH)), 4.23–4.22 (m, 2H, COOCH_2_), 4.02 (t, *^3^J* = 6.5 Hz, 2H, CH_2_CH_2_CH_3_), 3.82–3.20 (m, 10H, H_pip_ + CH_2_N_pip_), 1.77–1.72 (m, 2H, CH_2_CH_2_CH_3_), 0.98 (t, *^3^J* = 7.3 Hz, 3H, CH_3_); ^13^C-NMR (176.08 MHz, DMSO-*d*_6_) δ (ppm): 165.19, 162.72, 149.39, 131.56, 129.10, 121.46, 120.07, 115.96, 114.33, 69.32, 66.10, 63.36, 58.17, 52.03, 50.77, 45.32, 45.20, 21.85, 10.26; HR-MS: C_23_H_31_N_2_O_4_ [M − H]^−^ calculated 399.2283 *m*/*z*, found 399.2263 *m*/*z*.

*1-(2-Fluorophenyl)-4-{2-hydroxy-3-[(4-propoxybenzoyl)oxy]propyl}piperazinediium dichloride* (**4b**). Yield: 63%; R_f_: 0.89; R_f_(rev): 0.48; M.p.: 167–170 °C; HPLC pur. 98.78 (254.8 nm); ^1^H-NMR (700.25 MHz, DMSO-*d*_6_) δ: 10.87 (bs, 2H, NH^+^), 7.99 (d, *^3^J* = 8.9 Hz, 2H, ArCOO), 7.19–7.09 (m, 3H, Ar-F), 7.06–7.02 (m, 3H, ArO + Ar-F), 6.00 (s, 1H, OH), 4.50–4.47 (m, 1H, CH(OH)), 4.23–4.22 (m, 2H, COOCH_2_), 4.02 (t, *^3^J* = 6.6 Hz, 2H, CH_2_CH_2_CH_3_), 3.74–3.23 (m, 10H, H_pip_ + CH_2_N_pip_), 1.77–1.72 (m, 2H, CH_2_CH_2_CH_3_), 0.98 (t, *^3^J* = 7.4 Hz, 3H, CH_3_); ^13^C-NMR (176.08 MHz, DMSO-*d*_6_) δ: 165.14, 162.69, 154.79 (d, ^1^*J*_C-F_ = 244.5 Hz), 138.23 (d, ^2^*J*_C-F_ = 8.5 Hz), 131.53, 124.91 (d, ^3^*J*_C-F_ = 2.9 Hz), 123.31 (d, ^3^*J*_C-F_ = 7.8 Hz), 121.43, 119.52 (d, ^4^*J*_C-F_ = 1.1 Hz), 116.09 (d, ^2^*J*_C-F_ = 20.3 Hz), 114.30, 69.29, 66.05, 63.31, 58.29, 52.33, 51.05, 46.84, 46.69, 21.83, 10.24; HR-MS: C_23_H_30_FN_2_O_4_ [M − H]^−^ calculated 417.2190 *m*/*z*, found 417.2172 *m*/*z*.

*1-(4-Fluorophenyl)-4-{2-hydroxy-3-[(4-propoxybenzoyl)oxy]propyl}piperazinediium dichloride* (**4c**). Yield: 57%; R_f_: 0.85; R_f_(rev): 0.39; M.p.: 203–205 °C; HPLC pur. 99.33 (254.8 nm); ^1^H-NMR (700.25 MHz, DMSO-*d*_6_) δ: 10.80 (bs, 2H, NH^+^), 7.98 (d, *^3^J* = 8.9 Hz, 2H, ArCOO), 7.11–7.09 (m, 2H, Ar-F), 7.05–7.02 (m, 4H, ArO + Ar-F), 5.19 (s, 1H, OH), 4.50–4.47 (m, 1H, CH(OH)), 4.24–4.20 (m, 2H, COOCH_2_), 4.02 (t, *^3^J* = 6.5 Hz, 2H, CH_2_CH_2_CH_3_), 3.74–3.16 (m, 10H, H_pip_ + CH_2_N_pip_), 1.77–1.72 (m, 2H, CH_2_CH_2_CH_3_), 0.98 (t, *^3^J* = 7.4 Hz, 3H, CH_3_); ^13^C-NMR (176.08 MHz, DMSO-*d*_6_) δ: 165.15, 162.69, 156.55 (d, ^1^*J*_C-F_ = 236.9 Hz), 146.31 (d, ^4^*J*_C-F_ = 1.5 Hz), 131.53, 121.43, 117.77 (d, ^3^*J*_C-F_ = 7.6 Hz), 115.45 (d, ^2^*J*_C-F_ = 22.0 Hz), 114.30, 69.29, 66.07, 63.34, 58.12, 52.05, 50.78, 45.97, 45.85, 21.83, 10.24; HR-MS: C_23_H_30_FN_2_O_4_ [M − H]^−^ calculated 417.2190 *m*/*z*, found 417.2179 *m*/*z*.

*1-{2-Hydroxy-3-[(4-propoxybenzoyl)oxy]propyl}-4-(2-methoxyphenyl)piperazinediium dichloride* (**4d**). Yield: 56%; R_f_: 0.77; R_f_(rev): 0.51; M.p.: 127–130 °C; HPLC pur. 94.78 (254.8 nm); ^1^H-NMR (700.25 MHz, DMSO-*d*_6_) δ: 10.71 (bs, 2H, NH^+^), 7.99 (d, *^3^J* = 9.0 Hz, 2H, ArCOO), 7.04 (d, *^3^J* = 8.9 Hz, 2H, ArO), 7.03–7.02 (m, 1H, Ar), 6.99–6.98 (m, 1H, Ar), 6.96–6.95 (m, 1H, Ar), 6.92–6.90 (m, 1H, Ar), 5.11 (s, 1H, OH), 4.49–4.46 (m, 1H, CH(OH)), 4.24–4.21 (m, 2H, COOCH_2_), 4.02 (t, *^3^J* = 6.6 Hz, 2H, CH_2_CH_2_CH_3_), 3.80 (s, 3H, OCH_3_), 3.71–3.11 (m, 10H, H_pip_ + CH_2_N_pip_), 1.78–1.73 (m, 2H, CH_2_CH_2_CH_3_), 0.98 (t, *^3^J* = 7.4 Hz, 3H, CH_3_); ^13^C-NMR (176.08 MHz, DMSO-*d*_6_) δ: 165.14, 162.69, 151.78, 139.19, 131.53, 123.49, 121.43, 120.79, 118.24, 114.30, 111.97, 69.29, 66.03, 63.27, 58.30, 55.36, 52.58, 51.20, 46.76, 46.62, 21.82, 10.24; HR-MS: C_24_H_33_N_2_O_5_ [M − H]^−^ calculated 429.2389 *m*/*z*, found 429.2361 *m*/*z*.

*1-{2-Hydroxy-3-[(4-propoxybenzoyl)oxy]propyl}-4-(4-methoxyphenyl)piperazinediium dichloride* (**5e**). Yield: 63%; R_f_: 0.81; R_f_(rev): 0.49; M.p.: 191–194 °C; HPLC pur. 98.70 (254.8 nm); ^1^H-NMR: (700.25 MHz, DMSO-*d*_6_) δ: 10.85 (bs, 2H, NH^+^), 7.99 (d, *^3^J* = 7.9 Hz, 2H, ArCOO), 7.05–7.03 (m, 4H, Ar), 6.89 (d, *^3^J* = 8.2 Hz, 2H, NAr), 5.05 (s, 1H, OH), 4.49–4.47 (m, 1H, CH(OH)), 4.25–4.20 (m, 2H, COOCH_2_), 4.02 (t, *^3^J* = 6.5 Hz, 2H, CH_2_CH_2_CH_3_), 3.76–3.21 (m, 10H, H_pip_ + CH_2_N_pip_), 3.71 (s, 3H, OCH_3_), 1.77–1.72 (m, 2H, CH_2_CH_2_CH_3_), 0.98 (t, *^3^J* = 7.4 Hz, 3H, CH_3_); ^13^C-NMR (176.08 MHz, DMSO-*d*_6_) δ: 165.16, 162.69, 154.27, 142.65, 131.54, 121.43, 118.40, 114.46, 114.30, 69.30, 66.07, 63.38, 58.09, 55.24, 51.94, 50.70, 46.95, 46.86, 21.83, 10.24; HR-MS: C_24_H_33_N_2_O_5_ [M − H]^−^ calculated 429.2389 *m*/*z*, found 429.2364 *m*/*z*.

### 3.3. Quantum Chemical Calculations

The models of the complexes were prepared using interactive computer graphics, and their structures were optimized as follows. First, the harmonic vibrational frequencies were calculated employing the density functional theory (DFT)-based B3LYP/6-311G** method to assess the curvature of the potential-energy surface (PES). This served as an input for the gradient-based geometrical optimization at the same level of the quantum chemical theory. The resulting stationary points of the PES were then verified to be minima by calculating the harmonic vibrational frequencies again. The geometries thus obtained were used for the predictions of the NMR chemical shielding by combining the GIAO strategy [21,22] with the B3LYP/6-311G** method. This approach, further referred to as GIAO-B3LYP/6-311G**, was successfully applied to study the NMR parameters in peptides [14] and in boron-containing systems [23]. All the aforementioned calculations were carried out using the Gaussian 09 package [24].

## 4. Conclusions

In this study, five new potential dual β- and α_1_-blockers were prepared. These arylcarbonyloxyaminopropanols substituted by various phenylpiperazine moieties were designed as antihypertensive drugs with vasodilating properties and ultra-short effect. In this paper, the synthesis and characterisation of the compounds is discussed. When hydrogen chloride was added to the equimolar amount for preparation of the hydrochloride salt, it was determined by means of ^13^C-, ^15^N-CP/MAS NMR and IR that piperazine nitrogen substituted by aryl was protonated. When excess hydrogen chloride was used for preparation of salts, both nitrogen atoms in the piperazine ring were protonated.

## Figures and Tables

**Table 1:** Experimentally determined IR data of discussed compounds.

Compound	IR ν (cm^−1^)
**4a**	3319 (OH); 2336 (N-H^+^); 1704 (C=O); 1601, 1450 (aromatics); 1253, 1168 (ester)
**4b**	3290 (OH); 2528, 2445 (N-H^+^); 1701 (C=O); 1604, 1458 (aromatics); 1249, 1175 (ester)
**4c**	3285 (OH); 2503, 2401 (N-H^+^); 1712 (C=O); 1605, 1458 (aromatics); 1249, 1171 (ester)
**4d**	3318 (OH); 2349 (N-H^+^); 1706 (C=O); 1606, 1448 (aromatics); 1250, 1171 (ester)
**3e**	3071 (OH + CH); 1699 (C=O); 1604, 1439 (aromatics); 1255, 1163 (ester)
**4e**	3287 (OH); 2527, 2442 (N-H^+^); 1699 (C=O); 1603, 1462 (aromatics); 1248, 1171 (ester)
**5e**	3312 (OH); 2401 (N-H^+^); 1711 (C=O); 1604, 1462 (aromatics); 1250, 1171 (ester)

## References

[1] Prichard B.N.C., Graham B., Cruickshank J.M. (2001). β-Blockers in the third millennium—When are they really indicated?. J. Clin. Basic Cardiol..

[2] Roth H.J., Fenner H. (2000). Adrenozeptoren-Blocker (Sympatholytika). Arzneistoffe.

[3] Feuerstein G.Z., Ruffolo R.R. (1995). Carvedilol, a novel multiple action antihypertensive agent with antioxidant activity and the potential for myocardial and vascular protection. Eur. Heart J..

[4] Gupta S., Wright H.M. (2008). Nebivolol: A highly selective β1-adrenergic receptor blocker that causes vasodilatation by increasing nitric oxide. Cardiovasc. Ther..

[5] Wiest D. (1995). Esmolol. A review of its therapeutic efficiacy and pharmacokinetic characteristics. Clin. Pharmacokinet..

[6] Atarashi H., Kuruma A., Yashima M., Saitoh H., Ino T., Endoh Y., Hayakawa H. (2000). Pharmacokinetics of landiolol hydrochloride, a new ultra-short-acting β-blocker, in patients with cardiac arrhythmias. Clin. Pharmacol. Ther..

[7] Tengler J., Stropnicky O. (2014). Soft drugs and retrometabolic drug design. Chem. Listy.

[8] Bodor N., Buchwald P. (2000). Soft drug design: General principles and recent applications. Med. Res. Rev..

[9] Mokry P., Zemanova M., Csollei J., Racanska E., Tumova I. (2003). Synthesis and pharmacological evaluation of novel potential ultrashortacting β-blockers. Pharmazie.

[10] Bartosova L., Frydrych M., Hulakova G., Berankova K., Strnadova V., Mokry P., Brunclik V., Kolevska J., Bebarova M. (2004). Efficacy of newly synthesized 44Bu ultrashort-acting β-adrenergic antagonist to isoprenaline-induced tachycardia—Comparison with esmolol. Acta. Vet. Brno.

[11] Racanska E., Kurfurst P., Csollei J., Svec P. (2004). Cardiovascular effects of newly sythesized hybrid heteroarylaminoethanols. Acta Fac. Pharm. Univ. Comen..

[12] Ammazzalorso A., Amoroso R., Bettoni G., Fantacuzzi M., De Filippis B., Giampietro L., Maccallini C., Paludi D., Tricca M.L. (2004). Synthesis and antibacterial evaluation of oxazolidin-2-ones structurally related to linezolid. Farmaco.

[13] Tengler J., Kapustikova I., Stropnicky O., Mokry P., Oravec M., Csollei J., Jampilek J. (2013). Synthesis of new (arylcarbonyloxy)aminopropanol derivatives and the determination of their physico-chemical properties. Cent. Eur. J. Chem..

[14] Pawlak T., Trzeciak-Karlikowska K., Czernek J., Ciesielski W., Potrzebowski M.J. (2012). Computed and experimental chemical shift parameters for rigid and flexible YAF peptides in the solid state. J. Phys. Chem. B.

[15] Apperley D.C., Basford P.A., Dallman C.I., Harris R.K., Kinns M., Marshall P.V., Swanson A.G. (2005). Nuclear magnetic resonance investigation of the interaction of water vapor with sildenafil citrate in the solid state. J. Pharm. Sci..

[16] Wang Y., Wilson D., Harbison G.S. (2016). Solid-State NMR and the crystallization of aspartic and glutamic acids. Cryst. Growth Des..

[17] Policianova O., Brus J., Hruby M., Urbanova M., Zhigunov A., Kredatusova J., Kobera L. (2014). Structural diversity of solid dispersions of acetylsalicylic acid as seen by solid-state NMR. Mol. Pharm..

[18] Wu X.L., Burns S.T., Zilm K.W. (1994). Spectral editing in CPMAS NMR. Generating subspectra based on proton multiplicities. J. Magn. Reson. A.

[19] Brus J. (2000). Heating of samples induced by fast magic-angle spinning. Solid State Nucl. Magn. Reson..

[20] Proks V., Brus J., Pop-Georgievski O., Vecernikova E., Wisniewski W., Kotek J., Urbanova M., Rypacek F. (2013). Thermal-induced transformation of polydopamine structures: An efficient route for the stabilization of the polydopamine surfaces. Macromol. Chem. Phys..

[21] Ditchfield R. (1974). Self-consistent perturbation theory of diamagnetism. I. A gauge-invariant LCAO method for N.M.R. chemical shifts. Mol. Phys..

[22] Wolinski K., Hinton J.F., Pulay P. (1990). Efficient implementation of the gauge-independent atomic orbital method for NMR chemical shift calculations. J. Am. Chem Soc..

[23] Kobera L., Czernek J., Streckova M., Urbanova M., Abbrent S., Brus J. (2015). Structure and distribution of cross-links in boron-modified phenol–formaldehyde resins designed for soft magnetic composites: A multiple-quantum 11B–11B MAS NMR correlation spectroscopy study. Macromolecules.

[24] Frisch M.J., Trucks G.W., Schlegel H.B., Scuseria G.E., Robb M.A., Cheeseman J.R., Scalmani G., Barone V., Mennucci B., Petersson G.A. (2009). Gaussian 09, Revision D.01.

